# Enhanced survival in vitro of human corneal endothelial cells using mouse embryonic stem cell conditioned medium

**DOI:** 10.1167/2.7.611

**Published:** 2010-04-08

**Authors:** Xiaoyan Lu, Dong Chen, Zhiping Liu, Chaoyang Li, Ying Liu, Jin Zhou, Pengxia Wan, Yong-gao Mou, Zhichong Wang

**Affiliations:** 1 State Key laboratory of Ophthalmology, Sun Yat-sen University, Guangzhou, PR China; 2 Department of ENT, First Affiliated Hospital, Sun Yat-sen University, Guangzhou, PR China; 3 State Key Laboratory of Oncology in Southern China, Guangzhou, PR China; 4 Department of Neurosurgery, Cancer Center, Sun Yat-sen University, Guangzhou, PR China; 5 Centre for Stem Cell Biology and Tissue Engineering, Sun Yat-sen University, Guangzhou, PR China

## Abstract

**Purpose:**

To determine whether mouse embryonic stem cell conditioned medium (ESC-CM) increases the proliferative capacity of human corneal endothelial cells (HCECs) in vitro.

**Methods:**

Primary cultures of HCECs were established from explants of the endothelial cell layer, including the Descemet’s membrane. Cells were cultured in human corneal endothelium medium (CEM) containing 25% ESC-CM for the experimental group and CEM alone for the control group. Phase-contrast microscopy and reverse-transcription polymerase chain reaction (RT–PCR) were used to identify HCECs. The eruption time and HCEC morphology were observed under phase-contrast microscopy. We detected the protein expression of zona occludens protein-1 (ZO-1; a tight junction protein) and the Na^+^-K^+^-ATPase by western blot analysis and immunocytochemistry. The mRNA expression of the *Na^+^-K^+^-ATPase*, voltage-dependent anion channel 3 (*VDAC3*), solute carrier family 4, sodium bicarbonate cotransporter member 4 (*SLC4A4*), and chloride channel protein 3 (*CLCN3*) were detected by RT–PCR. To explore the proliferation capacity of HCECs, the colony forming efficiency (CFE) was determined by Giemsa staining and the cellular proliferation marker of Ki-67 protein (Ki-67) positive cells were detected by immunocytochemistry and flow cytometry. Progression of the cell cycle and apoptosis were analyzed by flow cytometry. Negative regulation of the cell cycle, as measured by cyclin-dependent kinase inhibitor p21 (p21) levels, was detected by western blot analysis and immunocytochemistry.

**Results:**

In primary culture, HCECs in the 25%ESC-CM group erupted with polygonal appearance on day 2, while those in the CEM group erupted with slightly larger cells on day 3–4. HCECs in the 25%ESC-CM group could be subcultured until passage 6 without enlargement of cell volume, while those in the CEM group were enlarged and lost their polygonal appearance by passage 2. HCECs in both the 25%ESC-CM and CEM groups expressed ZO-1, Na^+^-K^+^-ATPase, VDAC3, SLC4A4, and CLCN3. The number of Ki67 positive cells, CFE, and percentage of cells entering the S and G_2_ phases were higher in the 25%ESC-CM group than in the CEM group. The number of apoptotic cells and p21 protein expression both decreased in the 25%ESC-CM group.

**Conclusions:**

Use of 25%ESC-CM significantly increased the number of proliferating cells. These effects may be achieved through inhibition of p21 expression and apoptosis. These results suggested that 25%ESC-CM may be a new tool for cultivating HCECs for transplantation.

## Introduction

Human corneal endothelial cells (HCECs) are essential for maintaining corneal transparency via their pump and barrier functions. Since HCECs have poor proliferative potency in vivo, their associated disorders such as Fuchs’ endothelial dystrophy, pseudophakic bullous, and keratopathy, as well as trauma, can lead to a compensatory enlargement of the remaining endothelial cells and irreversible corneal endothelial cell dysfunction. Until now, corneal transplantation has been the most common treatment for corneal endothelial dysfunction. New surgical instruments and techniques have resulted in alternative methods for corneal endothelium surgery, including posterior lamellar keratoplasty, deep lamellar endothelial keratoplasty, and Descemet’s-stripping endothelial keratoplasty [1-3]. However, the global shortage of donor corneas remains acute, prompting efforts to produce donor corneas in vitro.

Recently, transplantation of cultivated HCECs has been suggested as an alternative treatment for corneal endothelial dysfunction [4,5]. However, HCECs are arrested at the G_1_ phase of the cell cycle in vivo [6-8]. Factors involved in this cell-cycle arrest include the relative lack of positive mitogenic stimulation in vivo [9,10], potential negative regulation by transforming growth factor-beta2 (TGF-beta2) in the aqueous humor [11-13], maintenance of the monolayer in a contact-inhibited state [14], and an age-dependent negative regulation [15-17]. Therefore, promoting HCECs from the G_1_ phase into the S phase is the most critical step in enhancing HCEC proliferation. Methods to enhance HCEC proliferation include: viral oncogene transformation of HCECs [12,18-21], addition of positive growth factors such as epidermal growth factor (EGF), basic fibroblast growth factor (bFGF), or nerve growth factor (NGF) to the culture medium [22], addition of an animal-derived extracellular matrix (ECM) [23-25], and addition of ethylenediaminetetraacetate acid (EDTA) to destroy the tight junctions between HCECs [25-29]. All these methods have limitations, including the long-term safety of transfected HCECs, the low efficiency and limited cell number of untransfected cultures, and the inability to subculture HCECs for more than a few passages [8]. Clearly, efficient culture techniques for HCECs require further development.

Embryonic stem cells (ESC) not only maintain their undifferentiated status and survive long-term, but they also promote proliferation, colony formation efficiency (CFE), and survival of other cells [30-34]. Liu et al. [35] found that treating HCECs with mouse embryonic stem cell conditioned medium (ESC-CM) significantly enhanced proliferation of rabbit corneal epithelial cells, rabbit conjunctival epithelial cells, cat corneal endothelial cells, and human epidermal cells. The present study was conducted to explore the capacity of ESC-CM to promote survival of HCECs by evaluating the morphology and biologic functions, including cell proliferation (CFE). This study also explores the probable mechanisms for how ESC-CM achieves its effects on HCECs.

## Methods

### Establishment of ESC-CM

Mouse ES-E14 cells were generously provided by Professor P. Xiang (Sun Yat-sen University, Guangzhou, China). The method to establish the ESC-CM was performed as described previously [35]. Briefly, mouse ES-E14 cells were plated at a density of 400/cm^2^ on 1% gelatin (Sigma-Aldrich, St. Louis, MO) coated tissue culture dishes containing mouse ES culture medium [36], with 50% of the medium changed and collected daily. The collected medium was added to the human corneal endothelium medium (CEM) to establish the ESC-CM system.

### Optimization of ESC-CM concentration

To compare the proliferation ability of the CEM group and the ESC-CM group, we needed to seed the same cell density in both groups. Using the passage 4 HCECs, we set up a density gradient from 5×10^3^ to 1×10^5^ cells/well and found that at a density of 9×10^4^ cells/well HCECs can growth well on 12-well plates in both groups. Then, we seeded passage 1 of HCECs at the density of 9×10^4^ cells/well on 12-wells in three different mediums: CEM (control I group), CEM containing ESC medium (ESC medium group, control II group) at the concentration of 10%, 25%, 35%, and 50% ESC medium, and in CEM containing ESC-CM (ESC-CM group, experimental group) at the concentration of 10%, 25%, 35%, and 50% ESC medium.

### Primary culture of HCECs

We obtained 24 human donor eyes following penetrating keratoplasty from the eye bank of Zhongshan Ophthalmic Center (Sun Yat-sen University, Guangzhou, China). The experiment was conducted according to the Declaration of Helsinki and was approved by the Ethics Committee of the Zhongshan Ophthalmic Center, Sun Yat-sen University. Donors were aged 21–68 years (35.6±11.2 years). The usage of the donor corneas was detailed in Table 1. To reduce the potential influence caused by donor tissues, we cut donor tissues equally into 2 parts for primary cultures in the CEM and ESC-CM groups. Every experiment was repeated more than three times in the same way. The corneas with cell density above 2,700–3,300 cells/mm^2^ were used for penetrating keratoplasty (PKP) within 24 h, the corneoscleral limbus with cornea endothelium left from the operation was stored in storage medium (Optisol; Bausch & Lomb Inc., Rochester, NY). Only the corneoscleral limbus with a cell density above 1,000 cells/mm^2^ was used for the primary culture within 12 h.

**Table 1:** The donor age and the number of corneas used in each experiment.

**Experiment**	**Corneas (n)**	**Age (years)**
Ki67(flow cytometry)	3	21,42.5,38
Cell cycle(flow cytometry)	6	23,30,32.5,35,43,56.5
Apoptosis(flow cytometry)	3	28,34,42.5
Weston Blot	3	29,33,35.5
Immunocytochemistry	2	28,32.5
RT-PCR	2	25,34
The concentration of screening	5	21,31.5,34.5,56,68

The left corneoscleral limbus obtained after the PKP operation was washed three times with OPTIMEM-1 (Invirogen-Gibco, Carlsbad, CA) containing 50 mg/ml gentamicin and 1.25 mg/ml amphotericin B. Then the left corneoscleral limbus obtained after the PKP operation was divided equally into two parts and the corneal endothelial cells with Descemet’s membrane were stripped from the posterior surface of the corneal tissue with sterile surgical forceps under a dissecting microscope. The explants were shredded into small pieces, about 50 pieces available for one part. The small pieces were placed into 12-well plates containing culture medium. The dishes were placed in an incubator at 37 °C. After four days, the culture medium was changed and was replaced every other day thereafter.

Primary cultures of HCECs in CEM were maintained as described by Chen et al. [22]. Briefly, the CEM contained OPTIMEM-1 as a basal medium, 8% fetal bovine serum (Invirogen-Gibco), 40 ng/ml fibroblast growth factor (FGF), 5 ng/ml epidermal growth factor (EGF), 20 ng/ml nerve growth factor (NGF), 20 µg/ml ascorbic acid, 0.005% human lipids, 200 mg/l calcium chloride, 0.08% chondroitin sulfate, 1% RPMI-1640 multiple vitamin solution, 50 µg/ml gentamicin, and antibiotic/antimycotic solution (diluted 1/100; all from Sigma-Aldrich, St. Louis, MO). Cells were subcultured after reaching confluence by treating with trypsin/EDTA and seeded at a density of 9×10^4^ cells/well on 12-well plates.

### HCEC identification

HCECs were identified as described previously by Chen et al. [22]. Specifically, passage 4 HCECs were different from human corneal stromal fibroblasts and human corneal epithelial cells morphologically via phase-contrast microscopy of confluent cells. They were different by reverse transcription-polymerase chain reaction (RT–PCR) detection of mRNA (mRNA) for collagen VIII (*COL8A1*; a component of Descemet’s membrane secreted by endothelial cells) and neuron-specific enolase (*NSE*; a substance specific for endothelial cells), Vimentin (*Vim*; a protein specific for human corneal stromal fibroblasts), keratin 3 (*K3*; a cytokeratin specific for corneal epithelial cells).

### Gene expression analysis of HCECs

The method was performed as previously described by Chen et al. [22]. In detail, total RNA was isolated from passage 4 HCECs in CEM and 25%ESC-CM (n=3) with Trizol reagent (Invitrogen, Carlsbad, CA) according to the manufacturer’s instructions, and then quantified by absorption at 260 nm. Expression of mRNA was detected by semiquantitative RT–PCR with a housekeeping gene, glyceraldehyde-3-phosphatedehydro-genase (*GAPDH*), as an internal control. PCR was performed using specific primer pairs (Table 2) and PCR mix (Invitrogen) in a thermal cycler.

**Table 2:** Primer pairs used in reverse transcription-polymerase chain reaction detection. Keratin 3 (*K3*), neuron-specific enolase (*NSE), VDAC3, CLCN3, SLC4A4, and Na^+^-K^+^ -ATPase* were the fluid transport association protein of the corneal endothelium.

**Gene**	**Sense primer(5′-3′)**	**Anti-sense primer(3′-5′)**	**Tm (°C)**	**Length (bp)**
*K3*	GGCAGAGATCGAGGGTGTC	GTCATCCTTCGCCTGCTGTAG	56.00	398.00
*Vimentin*	ATGCTTCTTTGGCACGTCTTGACCT	ACTGCACCTGTCTCCGGTATTCGTT	62.00	395.00
*NSE*	GACAAACAGCGTTACTTAGGCAA	TCGCATGGCATCCCGAAAGC	60.00	393.00
*Collagen VIII*	ATGTGATGGCTGTGCTGCCT	CTCTTGGGCCAGGCTCTCCA	62.00	408.00
*Na+/K+ -ATPase*	GGATGATCATAAACTTAGCCTTG	TATAAGCCAAGAAACAAAGAATCG	56.00	224.00
*CLCN3*	GTCCATCAATCCATTTGGTAACA	ATAATGACTTCCAGAACGGGATA	57.00	207.00
*SLC4A4*	GTTCAGATGAATGGGGATACGC	CGAGCATAAACACAAAGCGTAA	58.00	243.00
*VDAC3*	CAGACCCTTCGACCAGGAGT	TTCGCAACCCCTAGACTTCAG	61.00	248.00
*GAPDH*	GCCAAGGTCATCCATGACAAC	GTCCACCACCCTGTTGCTGTA	65.00	498.00
*ACTB*	GCACCACTGGTATTGTCATGGA	ATCTTCATGAGGTAGTCCGTCA	64.00	156.00

### Immunocytochemistry

Passage 4 HCECs grown on glass coverslips were fixed with 4% paraformaldehyde for 20 min and then processed by standard immunofluorescence staining. The primary antibodies were mouse anti-ZO-1 (1:50; Abcam, Cambridge, UK), anti-Na^+^-K^+^-ATPase (1:200; Abcam), rabbit anti-Ki67 (1:200; Abcam), and anti-p21 (1:200; Santa Cruz Biotechnology, Inc., Santa Cruz, CA). The secondary antibodies were Alexa Flour® conjugated goat-anti-mouse IgG (1:200; Invitrogen) or FITC-conjugated donkey-anti-rabbit IgG (1:200; Invitrogen). Nuclei were counterstained with Hoechst 33342 (1:2000; Invitrogen) and examined with a laser scanning confocal microscope (LSM 510 META; Carl Zeiss, Oberkochen, Germany).

### Flow cytometry analyses of the cellular proliferation marker Ki-67 protein (Ki-67), the cell cycle, and apoptotic/necrotic cell death

HCECs were dissociated into single cells by trypsin/EDTA digestion. For testing Ki67 positive cells, we digested the passage 4 HCECs and fixed them in stationary liquid (Invitrogen). For examining the cell cycle, passage 2 and passage 4 cells were washed and resuspended in −20 °C alcohol and then stored at −4 °C overnight before flow cytometry analysis [37,38]. To examine apoptotic/necrotic cell death, passage 4 HCECs were centrifuged and washed in cold PBS. HCECs were treated with Annexin V-FITC (Invitrogen) and stained with propidium iodide (PI; Invitrogen) and then resuspended in binding buffer at a concentration of 5×10^5^ cells/ml. Data analysis was conducted using CellQuest software (BD Biosciences, Mountain View, CA) [39,40].

### Western blot analysis

Passage 4 HCECs were removed from the culture plate 1 day after reaching confluence in both the CEM group and 25% ESC-CM group and then used to detect Na^+^-K^+^-ATPase and p21 protein expression. The primary antibodies used were mouse anti-Na^+^-K^+^-ATPase (1:1000; Abcam) and anti-p21 (1:1000; Santa Cruz Biotechnology, Inc.). The secondary antibodies used were Alexa Flour® conjugated goat-anti-mouse IgG (1:200; Invitrogen) or FITC-conjugated donkey-anti-rabbit IgG (1:200; Invitrogen) [41].

### CFE assay

CFE was determined as previously reported [42]. Briefly, passage 1 HCECs from the CEM group and 25% ESC-CM group were seeded at a density of 2×10^3^ cells/cm^2^ on 96-well plates (n=5) and cultured for 11 days, with the medium changed every other day. Then, the cells were stained with Giemsa (Sigma-Aldrich, St. Louis, MO). The number of colonies (at least 8 cells) was counted to determine the CFE (CFE=number of colonies/2,000×100%).

### Statistic analysis

**Figure 1:**
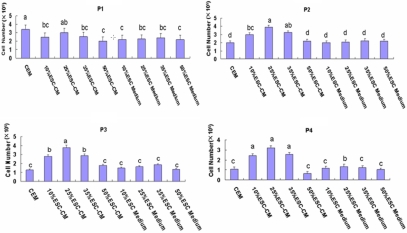
Cell number in three different mediums. The HCECs were seeded at the density of 9×10^4^ cells/well on 12-well plates. The ESC-CM and ESC medium were added to passage 1 cells. Data are expressed as the mean±SEM (n=3). The values with the same letter (a, b, c, d) in each column indicate these values are not significantly different (p>0.05). The same indication is used in all subsequent figures.

The statistical significance (p) of positive staining rate of Ki67, cell colony formation efficiency, the percentage of entering the cell cycle, and apoptosis rate was determined with the χ^2^ test. One-way analysis-of-variance to compare the p value of 9 groups and the Bonferroni Method for Multiple Comparisons to compare the data between each of the two groups in Figure 1. Values shown on graphs represent the mean±standard error, and a probability of 0.05 was considered statistically significant.

## Results

### The most effective concentration of ESC-CM

In preliminary experiments, we added ESC-CM and ESC medium to HCECs at passage 1. When HCECs reaching confluence, they were digested, and the number of cells were counted in each of the three groups (ESC-CM, ESC medium, and CEM). We found that passage 1 HCECs in different concentrations of ESC-CM and ESC medium showed transient cell death. From passage 2 to 4, the ESC-CM group had significantly more cells than either the CEM or ESC medium groups. Cell numbers were similar for the CEM and ESC medium groups. Among the ESC-CM groups, the concentrations of 10% and 35% had similar results, while the 25% group had the most cells and the 50% group had the least (Figure 1). Based on these results, we used 25% ESC-CM (25%ESC-CM) as the concentration in which to treat HCECs from the primary culture.

### Cell identification

**Figure 2:**
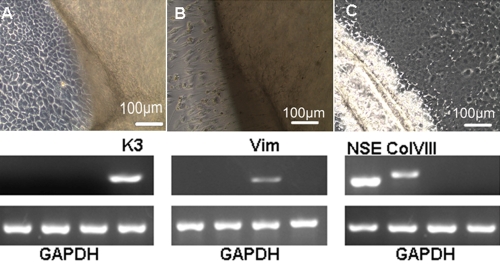
Cell identification results by phase-contrast microscopy and RT–PCR. **A**: Confluent cultures of human corneal epithelial cells expressing K3 (keratin 3). **B**: Human corneal stromal fibroblast cells expressing Vim (vimentin). **C**: HCECs expressing Col VIII (collagen VIII) and NSE (neuron-specific enolase).

Under phase-contrast microscopy, confluent cultured HCECs were rounder and smaller and expressed K3, a cytokeratin specific for corneal epithelial cells. Human corneal stromal fibroblasts exhibited a spindle-shape and characteristic “swirl” formation when reaching confluence and expressed Vim. HCECs had a “slabstone” appearance and expressed Col VIII and NSE (Figure 2).

### ESC-CM helped maintain the morphology and cell size of HCECs

**Figure 3:**
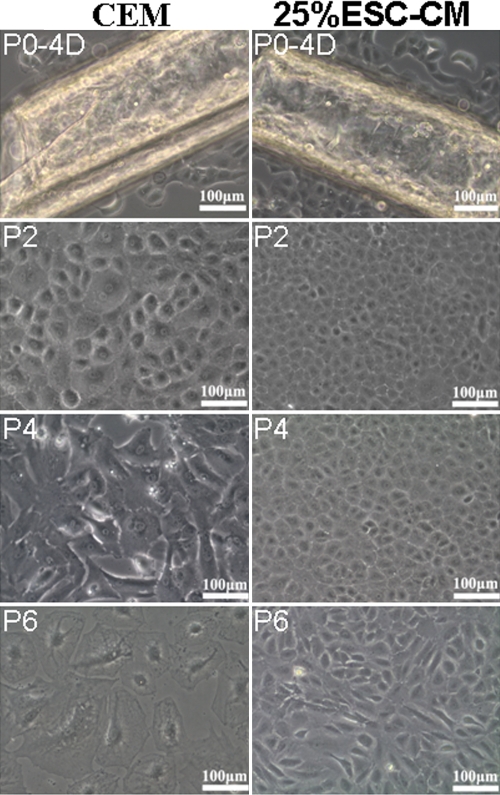
The morphology and cell size of HCECs cultured in the CEM group and the 25%ESC-CM group. The 25%ESC-CM group maintained the morphology and cell size of HCECs until passage (P6).

Explants of the endothelial cell layer adhered to the culture plate on day 2 in all groups. HCECs in 25%ESC-CM grew from the explants of the endothelial cell layer at day 2, while those in the CEM group grew from the explants of the endothelial cell layer at day 4. HCECs in the 25%ESC-CM group reached confluence at about day 10, while those in the CEM group took until day 14. In subculture, the 25%ESC-CM group reached confluence at 2–3 days while the CEM group needed 4–5 days. The morphology of the HCECs in 25%ESC-CM was hexagonal “slabstone” and remained the normal cell size of HCECs until passage 6. The HCECs in the CEM group got bigger at passage 2 and enlarged with each passage (Figure 3).

### ESC-CM treated HCECs retained the protein expression of ion channels, ZO-1 and Na^+^-K^+^-ATPase

**Figure 4:**
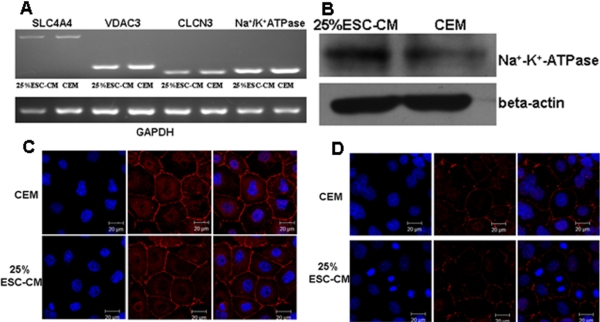
Expression of *VDAC3*, *CLCN3*, *SLC4A4*, and *Na^+^-K^+^-ATPase* by RT–PCR, and the protein expression of ZO-1 and Na^+^-K^+^-ATPase by immunocytochemistry. **A**: RT–PCR detection of the mRNA expression of *VDAC3*, *CLCN3*, *SLC4A4*, and *Na^+^-K^+^-ATPase*. **B**: western blot analysis of Na^+^-K^+^-ATPase . **C**: Immunostaining of ZO-1. **D**: Immunostaining of Na^+^-K^+^-ATPase.

The mRNA of *VDAC3*, *CLCN3*, *SLC4A4*, and *Na^+^-K^+^-ATPase* were expressed in HCECs (passage 4) in both the 25%ESC-CM and CEM groups (Figure 4A). Meanwhile, western blot analysis indicated that HCECs in both the 25%ESC-CM group and the CEM group expressed Na^+^-K^+^-ATPase (Figure 4B). Immunostaining for ZO-1 (Figure 4C) revealed that it was located at the cell boundaries of the cultivated HCECs in both the CEM group and the ESC-CM group. Na^+^-K^+^-ATPase (Figure 4D), an integral membrane protein complex responsible for regulating pump functions, was located at the basolateral membrane of the HCECs in both the CEM group and the ESC-CM group.

### ESC-CM enhanced proliferation of HCECs

**Figure 5:**
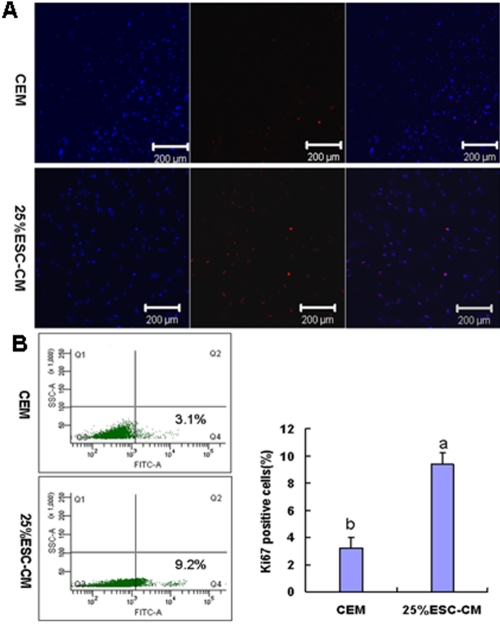
Increased frequency of proliferating HCECs in conditioned medium from mouse embryonic stem cells. **A**: Passage 4 HCECs were cultured for 48 h and probed for Ki67 expression. **B**: Ki67-positive cells were analyzed by flow cytometry. Flow cytometry analysis revealed significantly increased Ki67-positive HCECs in the 25%ESC-CM group (p=0.000). Data are expressed as the mean**±**SEM (n=3).

Expression of Ki67 was established to determine the role of 25%ESC-CM in the cell-cycle progression of HCECs. First, we used immunostaining with the cell-cycle population marker Ki67. At passage 4 in the 25%ESC-CM group, HCECs showed a higher number of Ki67-positive cells relative to the CEM group (Figure 5A). Further quantitative flow cytometry analysis revealed significantly increased Ki67-positive HCECs in the 25%ESC-CM group (p=0.000). Ki67-positive cells were higher 9.4±0.3% (n=3) in the 25%ESC-CM group than 3.2±0.3% (n=3) in the CEM group(Figure 5B).

### ESC-CM stimulated colony formation of HCECs

**Figure 6:**
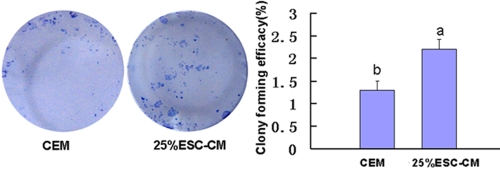
Conditioned medium from mouse embryonic stem cells stimulated colony formation of HCECs. The colony formation was significantly higher in the 25%ESC-CM group than in the CEM group (p=0.000). Data are expressed as the mean**±**SEM (n=5).

Passage 1 HCECs were used to detect colony formation and CEF. We observed more colonies in the 25%ESC-CM group than in the CEM group. The CEFs of the CEM and 25%ESC-CM groups were 1.3±0.7% (n=5) and 2.2±0.8%(n=5), respectively, and the colony formation of 25%ESC-CM groups was significantly higher than the CEM group(p=0.000; Figure 6).

### ESC-CM promoted cell-cycle entrance of HCECs

**Figure 7:**
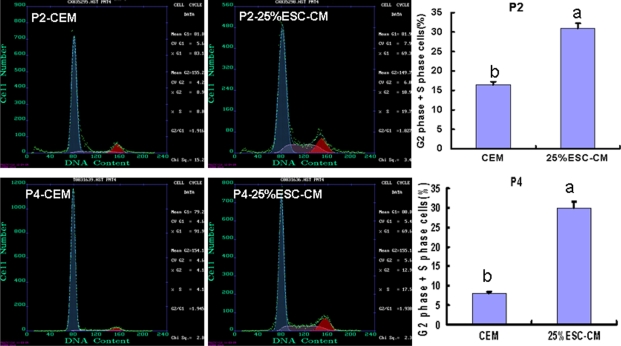
Conditioned medium from mouse embryonic stem cells promoted cell-cycle entrance of HCECs. The percentage of S phase and G_2_ phase cells in 25%ESC-CM group was significantly increased at passage 2 (p*=*0.001) and passage 4 (p=0.000). Data are expressed as the mean**±**SEM (n=3).

To detect the effect of 25%ESC-CM on the cell cycle, we examined the cell-cycle entrance of passage 2 and passage 4 HCECs from the 25%ESC-CM and CEM groups by flow cytometry. The cell-cycle entrance of HCECs treated with 25%ESC-CM was significantly higher than that of CEM group both in passage 2 (p=0.001) and passage 4 (p=0.000). The percentage of cells entering the S phase and G_2_ phase in the 25%ESC-CM and CEM groups were 30.9±1.3% (n=3) and 16.4±0.8% (n=3) in passage 2, respectively, while in passage 4 they were 29.9±1.7% (n=3) and 8.1±0.2% (n=3), respectively (Figure 7).

### ESC-CM inhibited HCECs apoptosis

**Figure 8:**
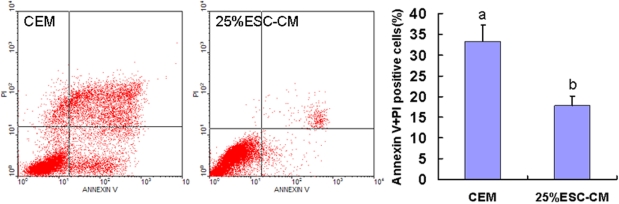
Conditioned medium from mouse embryonic stem cells inhibited the apoptosis of HCECs. The apoptosis/necrosis rate of passage 4 HCECs in 25%ESC-CM group was significantly lower than in CEM group (p=0.001). Data are expressed as the mean**±**SEM (n=3).

We examined apoptosis and necrosis of cells at passage 4 in the 25%ESC-CM and CEM groups by flow cytometry. The flow cytometry revealed that 25%ESC-CM treatment significantly decreased apoptosis (p=0.001). The inhibition of apoptosis during subculture was lowered from 33.3±8.9% (n=3) in the CEM group to 17.9±7.7% (n=3) in the 25%ESC-CM group, thus implicating contribution of the anti-apoptosis of the 25%ESC-CM to the subculture of HCECs (Figure 8).

### ESC-CM inhibited p21 expression

**Figure 9:**
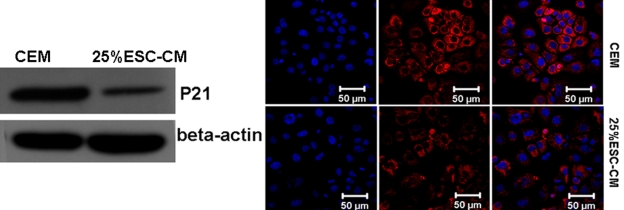
Conditioned medium from mouse embryonic stem cells inhibited the expression of p21. **A**: western blot analysis. **B**: Immunocytochemistry.

p21 expression decreased in the 25%ESC-CM group, as shown by western blot analysis (Figure 9A) and immunocytochemistry (Figure 9B).

## Discussion

In this study, we analyzed the effects of ESC-CM on the cellular phenotype, biologic function, proliferation capacity, cell cycle, and apoptosis of HCECs. The results showed that HCECs cultured in 25%ESC-CM group maintained normal cell size and appearance until passage 6 and expressed the same levels of ZO-1, Na^+^-K^+^-ATPase, VDAC3, SLC4A4, and CLCN3 as those cultured in the CEM group. Results showed that 25%ESC-CM significantly enhanced HCEC proliferation, promoted HCECs into the cell cycle, decreased apoptosis and necrosis, and downregulated the expression of the cell-cycle related protein p21.

In preliminary experiments, passage 1 HCECs cultured in different concentrations of ESC-CM or ESC medium showed transient cell death, perhaps because of the sudden change in the culture environment. In the following experiment, we started treating the HCECs in the primary culture. The proliferation capacity of HCECs at passages 2–4 in the ESC medium group and CEM group did not differ significantly in the preliminary experiment, indicating that the ESC medium had no significant effect on HCECs proliferation. This confirmed that the enhanced proliferation was the results of adding ESC-CM. Adding 10%ESC-CM and 35%ESC-CM had similar results, while 50%ESC-CM generated the least number of cells, demonstrating that except for the positive factors which to enhance proliferation, the ESC-CM also had the metabolic waste of murine ES cells. Further studies are needed to discover the key factors that enhance the proliferation and prevent apoptosis of HCECs in ESC-CM, so as to avoid the negative side effects of ESC-CM.

Cytokines/growth modulators secreted by ESC included interleukins IL-1α, IL-10, and IL-11, colony stimulating factors M-CSF (macrophage-colony stimulating factor) and GM-CSF (granulocyte macrophage-colony stimulating factor), growth factors/growth modulators including EGF (epidermal growth factor), FGF-basic (fibroblast growth factor-basic), FGF-9 (fibroblast growth factor-9), OSM (oncostatin M), SCF (stem cell factor), VEGF (vascular endothelial cell growth factor), IFN-γ (interferon-γ), insulin, LIF (leukemia inhibitory factor), and TNF-α (tumor necrosis factor-α), as well as several chemokines and other proteins, some of which are known to enhance the survival and/or prevent apoptosis of progenitor cells. Studies have shown that growth factors within the culture media of ESC are important, not only for maintaining their undifferentiated status and long-term survival, but also for promoting proliferation, CFE, and survival of other cells [30-34]. Our data indicated fewer apoptosis positive HCECs when treated with ESC-CM compared to CEM. The CFE observed was higher in the ESC-CM group relative to the CEM group, a result supported by other studies. This study for the first time discovered a phenomenon that the ESC-CM significantly promotes human corneal endothelial cell proliferation. We do not currently know the exact factors contained in the ESC-CM that promoted the proliferative capacity of HCECs. As such, further studies are planned to explore the effective molecules and mechanism in the near future.

p21 is a potent, tight-binding inhibitor of cyclin-dependent protein kinases (Cdks) that can inhibit the phosphorylation of Rb by cyclin A-Cdk2, cyclin E-Cdk2, cyclin D1-Cdk4, and cyclin D2-Cdk4 complexes. Furthermore, overexpression of p21 inhibits the proliferation of mammalian cells [43,44]. Bedelbaeva et al. [45] indicated that lack of p21 expression links cell cycle control and appendage regeneration in mice. Enomoto et al. [15] also observed an age-dependent increase in negative regulation of the cell cycle by p21. Transforming growth factor beta (TGF-beta) is a group of multifunctional growth factors that inhibit cell-cycle progression in many cell types [46], including HCECs [47]. Datto et al. [48] demonstrated that TGF-beta also causes a rapid transcriptional induction of p21, suggesting that p21 can respond to both intracellular and extracellular signals for cell-cycle arrest. Xiao et al. [49] proposed that the p53/p21 (WAF1/CIP1) signaling pathway may exert its principle effect during the late stages of senescence in corneal endothelium cells. Studies indicate that the multiple functions of p21 are associated with its location within the cell. When expressed in the cytoplasm, p21 enhances cell-cycle entrance and prevents apoptosis [50-52]. Our results indicated that 25%ESC-CM treated HCECs expressed lower protein levels of p21 than those treated with CEM. We consider it reasonable to suspect that 25%ESC-CM may promote the cell-cycle entrance of HCECs, at least in part, by downregulating p21 expression.

Although all experiments in this study were repeated at least three times, the results may have limitation to the conditions tested. Further investigations are necessary to develop optimal conditions for corneal endothelial cultures.

In this experiment, ESC-CM is derived from mouse ES cell. The components of animal origin may cause a health risk in cell therapy and other clinical applications. Thus, identification of effective factors in the mouse ESC-CM and investigation of the effects of human ES cells on corneal endothelial proliferation are important steps in our future studies.

Although the underlying mechanisms have yet to be revealed, murine ESC-CM clearly promoted the cell-cycle progression of HCECs. Further investigations are necessary to determine the mechanisms underlying the increased cell proliferation observed in our studies.

In summary, our findings demonstrated that 25%ESC-CM significantly enhanced the proliferative capacity of HCECs and also maintained the morphology, cell size, and biologic functions of these cells. This enhanced proliferative capacity is achieved through promotion of colony formation, inhibition of p21 expression, and decreased apoptosis.
